# Complete response of metastatic renal cell carcinoma with inferior vena cava tumor thrombus to nivolumab plus ipilimumab

**DOI:** 10.1002/iju5.12452

**Published:** 2022-04-26

**Authors:** Michikata Hayashida, Yuji Miura, Taisho Noda, Taro Yamanaka, Naoto Tanaka, Shotaro Yasuoka, Suguru Oka, Kazushige Sakaguchi, Keiichi Kinowaki, Shinji Urakami

**Affiliations:** ^1^ Departments of Urology Toranomon Hospital Tokyo Japan; ^2^ Department of Medical Oncology Toranomon Hospital Tokyo Japan; ^3^ Department of Pathology Toranomon Hospital Tokyo Japan

**Keywords:** inferior vena cava tumor thrombus, metastatic renal cell carcinoma, nivolumab plus ipilimumab

## Abstract

**Introduction:**

The effectiveness of nivolumab plus ipilimumab for metastatic renal cell carcinoma with inferior vena cava tumor thrombus remains unclear.

**Case presentation:**

A 75‐year‐old male was diagnosed with metastatic renal cell carcinoma with inferior vena cava tumor thrombus and treated with nivolumab plus ipilimumab. The renal mass and thrombus regressed and all pulmonary nodules except for one lesion diminished. To avoid thrombotic complications, radical nephrectomy and thrombectomy were performed. No viable malignant cells were revealed histopathologically. Although nivolumab was continued after the surgical interventions, the remaining lesion did not change. Considering the discontinuation of nivolumab, metastasectomy was performed, and no viable malignant cells were revealed histopathologically. There has been no recurrence after the discontinuation.

**Conclusion:**

Nivolumab plus ipilimumab could have effectiveness for metastatic renal cell carcinoma with inferior vena cava tumor thrombus.

**Abbreviations & Acronyms**
CR: complete response
CT: computed tomography
ICI: immune checkpoint inhibitor
IMDC: International Metastatic RCC Database Consortium
IVC: inferior vena cava
IVCTT: inferior vena cava tumor thrombus
mRCC: metastatic renal cell carcinoma
MRI: magnetic resonance imaging
Nivo‐Ipi: nivolumab plus ipilimumab
OS: overall survival

**Keynote message**

Nivolumab plus ipilimumab could have effectiveness for metastatic renal cell carcinoma with inferior vena cava tumor thrombus.

## Introduction

Nivo‐Ipi has been a standard first‐line treatment for mRCC with IMDC intermediate and poor risk. We encountered a patient who achieved cancer‐free status with Nivo‐Ipi and surgical resection for mRCC with IVCTT. We report this clinical course as it may help urologists decide on treatment for mRCC with IVCTT.

## Case presentation

**Fig. 1:**
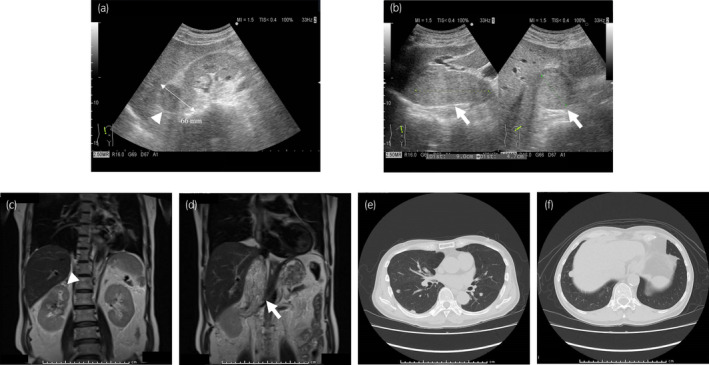
Images of the renal tumor in the upper portion of the right kidney and IVCTT. (a, b) Ultrasonography images. Ultrasonography revealed a 66 mm renal mass in the upper portion of the right kidney and a tumor thrombus within the IVC, the major axis and diameter of which is 90 and 47 mm, respectively. (c, d) T2‐weighted coronary images of MRI. MRI showed a renal mass at the upper pole of the right kidney and a tumor thrombus that extended into the IVC and beyond the hepatic vein. (e, f) CT images. CT revealed multiple pulmonary masses suspected of being lung metastases. [Colour figure can be viewed at wileyonlinelibrary.com]

A 75‐year‐old male presented with shortness of breath. Physical examination showed no abnormal findings. Blood tests showed renal function degeneracy. Ultrasonography for screening of post‐renal dysfunction showed a 66 mm mass in the upper portion of the right kidney and IVCTT (Fig. 1a,b). MRI showed IVCTT, with a major axis of 90 mm and maximum diameter of 47 mm, extended beyond the hepatic vein (Neves classification level III),^1^ causing dilation of the IVC (Fig. 1c,d). CT revealed multiple pulmonary nodules, and no obvious lymphadenopathy was observed (Fig. 1e,f). Percutaneous needle biopsy for the renal mass demonstrated clear cell renal cell carcinoma with abundant infiltrating immune cells. Therefore, the patient was diagnosed with mRCC with IVCTT. His IMDC risk was intermediate, given the anemia and time from diagnosis to systemic therapy.

**Fig. 2:**
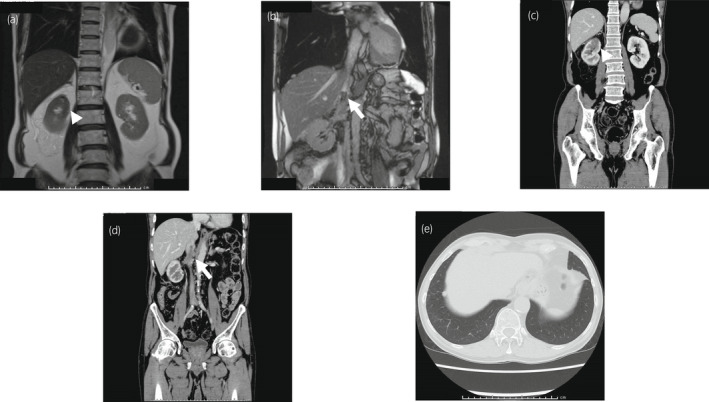
Images of the renal tumor and IVCTT after four cycles of Nivo‐Ipi and four cycles of nivolumab monotherapy. (a, b) T2‐weighted coronary images of MRI. (c, d) Images of contrast‐enhanced CT. (e) A CT image of the pulmonary metastatic lesion. The renal mass and IVCTT had regressed and multiple pulmonary masses had diminished, except for one lesion.

**Fig. 3:**
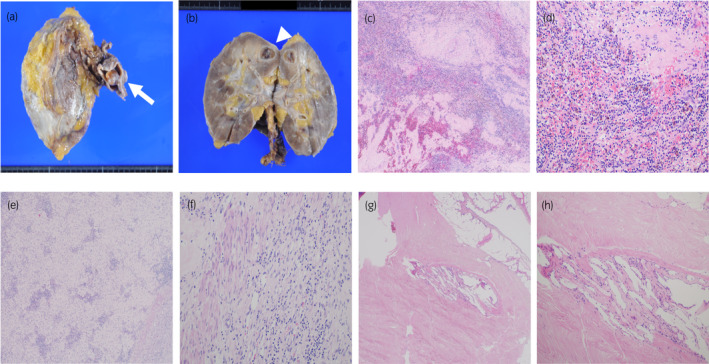
Images of histopathological examination. (a, b) Macroscopic images of the resected specimen (the renal mass and the tumor thrombus). The tumor thrombus (white arrow) was extended from the renal vein into the IVC and a white renal mass (arrowhead) with unclear boundaries in the upper portion of the right kidney was observed. Microscopic images of hematoxylin and eosin staining of the specimen are presented in panels c–h. (c, d) Microscopic images of the renal mass in 40× and 200× fields of view. (e, f) Microscopic images of the tumor thrombus in 40× and 200× fields of view. Pathological examination revealed the existence of inflammation cells, mainly lymphocytes, and there were no viable malignant cells. (g, h) Microscopic images of the remaining pulmonary metastatic lesion in 40× and 100× fields of view. Pathological examination showed fibrosis without viable malignant cells. [Colour figure can be viewed at wileyonlinelibrary.com]

He received four cycles of Nivo‐Ipi followed by four cycles of nivolumab monotherapy. CT and MRI revealed the IVCTT had regressed to the IVC beneath the liver and the dilation of the IVC was resolved (Fig. 2a–d). Furthermore, CT showed the lung metastases had diminished, except for one lesion at the bottom of the right lung (Fig. 2e). Given that his performance status was good and a resection of the IVCTT could be safer since clamping the IVC to avoid intraoperative thrombotic complications was easier and the range of operation was smaller because the IVCTT had regressed, radical nephrectomy with removal of the IVCTT was performed to avoid cardiovascular events due to IVC obstruction and thrombotic complications. The IVCTT was completely removed but resection of the IVC and reconstruction with a vascular graft was needed since the adhesions to the IVC wall were too tight to separate. The resected specimens showed a white tumor with unclear boundaries in the upper portion of the right kidney and infiltration of the tumor into the IVC from the renal vein (Fig. 3a,b). However, no viable malignant cells were revealed histopathologically (Fig. 3c–f). Although seven cycles of nivolumab monotherapy were continued after the operation, the remaining pulmonary lesion did not change. To judge whether nivolumab monotherapy could be suspended or not, metastasectomy of the pulmonary lesion was performed. Pathological examination showed fibrosis without viable malignant cells (Fig. 3g,h), suggesting the patient could achieve CR of mRCC with IVCTT to Nivo‐Ipi. Nivolumab monotherapy was discontinued, given the pathological findings and the patient's request. There has been no recurrence for 14 months after the discontinuation.

## Discussion

The CheckMate 214 trial, which evaluated the efficacy of Nivo‐Ipi, demonstrated improvement in OS and the objective response rate in IMDC intermediate‐ and poor‐risk patients compared to sunitinib.^2^ However, it was unclear whether Nivo‐Ipi could have efficacy for mRCC with IVCTT, according to CheckMate 214 trial protocols.^2^ Only two CR cases of mRCC with IVCTT to Nivo‐Ipi have been reported^3^, ^4^ (Table 1). Therefore, the present case, in which the CR of mRCC with IVCTT to Nivo‐Ipi was confirmed pathologically, is valuable and suggests that Nivo‐Ipi could have effectiveness for mRCC with IVCTT.

**Table 1:** Details of reported CR cases of mRCC with IVCTT to Nivo‐Ipi. Information about each patient, primary tumor location, metastatic sites, pathological findings, and the clinical course is presented from present case and from two cases published in the literature

Author	Year	Sex	Age	Primary tumor	Level of IVCTT†	Metastatic sites	Pathological findings	Outcome
Okada *et al*.	2020	Male	47	Left middle pole 150 mm	Level III	Lung	CR in all lesions	No recurrence for 6 months after a discontinuation of systemic therapy
Shepherd *et al*.	2020	Male	61	Right lower pole 90 mm	Level III	Liver, lung, right acetabulum	CR in IVCTT. The primary tumor showed necrosis with residual viable cells focally seen	Discontinuation of systemic therapy. No information about recurrence.
Present case	2021	Male	75	Right upper pole 66 mm	Level III	Lung	CR in all lesions.	No recurrence for 14 months after discontinuation of systemic therapy

† Level of IVCTT is defined by Neves classification.

According to the previous two case reports, radical nephrectomy and thrombectomy were performed to obtain cancer‐free status after the disappearance of the metastatic sites. However, in this case, radical nephrectomy and thrombectomy were performed regardless of the solitary remaining pulmonary metastatic site, which meant cytoreductive surgery.

The CARMENA and SURTIME study, which prospectively evaluated the meaning of cytoreductive surgery for mRCC treated with tyrosine kinase inhibitors, showed no statistically significant results, suggesting that the indication and timing of cytoreductive surgery should be considered carefully.^5^, ^6^ Though one case study reported the effectiveness of cytoreductive surgery in mRCC cases treated with ICI, it remains unclear whether cytoreductive surgery plays an important role in mRCC treated with ICI.^7^, ^8^ The present case suggests cytoreductive surgery in mRCC with IVCTT can be meaningful in terms of avoiding cardiovascular and thrombotic complications that can cause sudden death. In addition, Nivo‐Ipi could have the potential to make cytoreductive surgery safer and easier through the regression of IVCTT. Therefore, in cases where the renal mass and IVCTT can be resected completely and safely after Nivo‐Ipi treatment, cytoreductive surgery should be considered to avoid fatal complications.

In this case, metastasectomy for the remaining pulmonary lesion in order to consider the discontinuation of nivolumab showed no viable malignant cells pathologically. This finding suggests that a difference between radiological and pathological response can exist after ICI treatment, which was also demonstrated by an earlier case report.^9^ Given this difference, metastasectomy in the ICI era may play an important role in considering treatment strategy by confirming pathological response. Furthermore, though most clinical evidence has come from small, retrospective single‐institutional studies, it has been suggested that complete metastasectomy could improve OS, especially in the cases of lung metastasis.^9^, ^10^, ^11^, ^12^ Therefore, if metastases remain shrunken and resectable solely with ICIs, metastasectomy could provide benefit in terms of both improving OS and considering therapeutic strategy.

## Conclusion

Nivo‐Ipi could have effectiveness for mRCC with IVCTT and the potential to reduce operative stress with a regression of the IVCTT. While it remains unclear whether cytoreductive surgery following ICI has meaning in mRCC cases, cytoreductive surgery for mRCC with IVCTT can avoid fatal complications. Metastasectomy in the ICI era may have an important role not only in improving OS but also in considering therapeutic strategy. Further studies with accumulated cases of mRCC with IVCTT treated with Nivo‐Ipi and surgical resection are needed to clarify the effectiveness of Nivo‐Ipi, the meaning of cytoreductive surgery, and the role of metastasectomy.

## Author Contributions

Michikata Hayashida: Conceptualization; data curation; formal analysis; methodology; project administration; resources; writing – original draft. Yuji Miura: Supervision; writing – review and editing. Taisho Noda: Data curation; investigation. Taro Yamanaka: Investigation; writing – review and editing. Naoto Tanaka: Data curation. Shotaro Yasuoka: Writing – review and editing. Suguru Oka: Writing – review and editing. Kazushige Sakaguchi: Writing – review and editing. Keiichi Kinowaki: Supervision. Shinji Urakami: Conceptualization; supervision.

## Conflict of interest

We have no conflict of interests to declare except for Yuji Miura. He has personal fees from Ono Pharmaceutical, Bristol Myers Squibb, MSD, and Takeda. However, all of them are outside of this case report.

## Approval of the research protocol by an Institutional Reviewer Board

Not applicable.

## Informed consent

Written informed consent was obtained.

## Registry and the Registration No. of the study/trial

Not applicable.

## References

[1] Neves RJ , Zincke H . Surgical treatment of renal cancer with vena cava extension. Br. J. Urol. 1987; 59: 390–5. 3594097 10.1111/j.1464-410x.1987.tb04832.x

[2] Motzer RJ , Tannir NM , McDermott DF *et al*. Nivolumab plus ipilimumab versus sunitinib in advanced renal‐cell carcinoma. N. Engl. J. Med. 2018; 378: 1277–90. 29562145 10.1056/NEJMoa1712126 PMC5972549

[3] Okada T , Hamamoto S , Etani T *et al*. Complete response of renal cell carcinoma with an inferior vena cava tumor thrombus and lung metastases after treatment with nivolumab plus ipilimumab. Int. Cancer Conf. J. 2020; 9: 88–91. 32257760 10.1007/s13691-020-00403-9 PMC7109205

[4] Shepherd ARH , Joshi R , Tan CP , Cohen P , Brook NR . Inferior vena cava thrombectomy following complete response to nivolumab/ipilimumab for metastatic renal cell carcinoma. ANZ J. Surg. 2020; 90: 1517–9. 31833177 10.1111/ans.15608

[5] Méjean A , Ravaud A , Thezenas S *et al*. Sunitinib alone or after nephrectomy in metastatic renal‐cell carcinoma. N. Engl. J. Med. 2018; 379: 417–27. 29860937 10.1056/NEJMoa1803675

[6] Bex A , Mulders P , Jewett M *et al*. Comparison of immediate vs deferred cytoreductive nephrectomy in patients with synchronous metastatic renal cell carcinoma receiving sunitinib: the SURTIME randomized clinical trial. JAMA Oncol. 2019; 5: 164–70. 30543350 10.1001/jamaoncol.2018.5543 PMC6439568

[7] Reimers MA , Figenshau RS , Kim EH *et al*. Cytoreductive nephrectomy after checkpoint inhibitor immunotherapy in patients with initially unresectable metastatic clear cell renal cell carcinoma. Clin. Genitourin. Cancer 2020; 18: 361–6. 32417157 10.1016/j.clgc.2020.04.002 PMC7541406

[8] Pignot G , Thiery‐Vuillemin A , Walz J *et al*. Nephrectomy after complete response to immune checkpoint inhibitors for metastatic renal cell carcinoma: a new surgical challenge? Eur. Urol. 2020; 77: 761–3. 31911011 10.1016/j.eururo.2019.12.018

[9] Hagimoto H , Kashima S , Doi K *et al*. Pathological complete response after nivolumab therapy following angiogenesis inhibitors in a patient with metastatic renal cell carcinoma. IJU Case Rep. 2020; 3: 287–90. 33163928 10.1002/iju5.12220 PMC7609183

[10] Adashek JJ , Aydin AM , Kim P , Spiess PE . The role of metastasectomy in the treatment of metastatic renal cell carcinoma. AME Med. J. 2019; 4: 30–41.

[11] Dabestani S , Marconi L , Hofmann F *et al*. Local treatments for metastases of renal cell carcinoma: a systematic review. Lancet Oncol. 2014; 15: e549–61. 25439697 10.1016/S1470-2045(14)70235-9

[12] Ouzaid I , Capitanio U , Staehler M *et al*. Surgical metastasectomy in renal cell carcinoma: a systematic review. Eur. Urol. Oncol. 2019; 2: 141–9. 31017089 10.1016/j.euo.2018.08.028

